# Transcriptome Analysis of the Oriental River Prawn, *Macrobrachium nipponense* Using 454 Pyrosequencing for Discovery of Genes and Markers

**DOI:** 10.1371/journal.pone.0039727

**Published:** 2012-06-20

**Authors:** Keyi Ma, Gaofeng Qiu, Jianbin Feng, Jiale Li

**Affiliations:** 1 Key laboratory of Freshwater Aquatic Genetic Resources Certificated by Ministry of Agriculture, College of Fisheries and Life Science, Shanghai Ocean University, Shanghai, P. R. China; 2 E-Institute of Shanghai Universities, Shanghai Ocean University, Shanghai, P. R. China; Auburn University, United States of America

## Abstract

**Background:**

The oriental river prawn, *Macrobrachium nipponense*, is an economically and nutritionally important species of the Palaemonidae family of decapod crustaceans. To date, the sequencing of its whole genome is unavailable as a non-model organism. Transcriptomic information is also scarce for this species. In this study, we performed *de novo* transcriptome sequencing to produce the first comprehensive expressed sequence tag (EST) dataset for *M. nipponense* using high-throughput sequencing technologies.

**Methodology and Principal Findings:**

Total RNA was isolated from eyestalk, gill, heart, ovary, testis, hepatopancreas, muscle, and embryos at the cleavage, gastrula, nauplius and zoea stages. Equal quantities of RNA from each tissue and stage were pooled to construct a cDNA library. Using 454 pyrosequencing technology, we generated a total of 984,204 high quality reads (338.59Mb) with an average length of 344 bp. Clustering and assembly of these reads produced a non-redundant set of 81,411 unique sequences, comprising 42,551 contigs and 38,860 singletons. All of the unique sequences were involved in the molecular function (30,425), cellular component (44,112) and biological process (67,679) categories by GO analysis. Potential genes and their functions were predicted by KEGG pathway mapping and COG analysis. Based on our sequence analysis and published literature, many putative genes involved in sex determination, including *DMRT1*, *FTZ-F1*, *FOXL2*, *FEM1* and other potentially important candidate genes, were identified for the first time in this prawn. Furthermore, 6,689 SSRs and 18,107 high-confidence SNPs were identified in this EST dataset.

**Conclusions:**

The transcriptome provides an invaluable new data for a functional genomics resource and future biological research in *M. nipponense*. The molecular markers identified in this study will provide a material basis for future genetic linkage and quantitative trait loci analyses, and will be essential for accelerating aquaculture breeding programs with this species.

## Introduction

The oriental river prawn *Macrobrachium nipponense*, a member of the Palaemonidae family of decapod crustaceans, is widely distributed in freshwater and low-salinity regions of estuaries [1]. This prawn is a useful model organism for studying reproduction and development in decapod species for multiple reasons. First, it produces large numbers of eggs due to its high fecundity. Each gravid female can lay thousands of eggs after mating. Second, *M. nipponense* is a relatively hardy species that adapts to a wide range of environmental conditions and is reared easily in a laboratory. Third, it can survive in freshwater for its entire life cycle. Hence, the costs are considerably lower than saltwater species. *M. nipponense* is also commercially important and is cultured extensively throughout China [2] and other Asian countries [3]–[7]. The basic production techniques for *M. nipponense* were developed in China approximately 40 years ago. In 2008, the culture yields of this prawn exceeded 205,000 tons, which accounts for the majority of its worldwide production. Owing to its high benefit and excellent adaptability, its culture production has gradually increased. However, due to the precocity of the farmed prawns, especially the females, the growth performance has decreased in recent years. On the other hand, there is a significant difference in growth performance between males and females in this species. Males grow much faster than females and reach a larger size at harvest, similarly to many other *Macrobrachium* species.

In monosex culture, energy from reproduction is diverted to growth, resulting in larger size of cultured individuals than that of mixed sex. Monosex culture is a common practice in aquaculture, and many attempts have been made to apply this technology to crustacean aquaculture [8]. The androgenic gland of crustaceans, an endocrine organ unique to males, plays a crucial role in the sexual differentiation to maleness [9]. Ablation or implantation of the androgenic gland at a certain stage of development could result in sex reversal to female or male, respectively [9]–[12]. Although all-male populations have been produced in *Macrobrachium rosenbergii* [8], corresponding research in *M. nipponense* has not been performed. Furthermore, the molecular mechanisms involved in sex determination/differentiation are poorly understood [13]. Due to a lack of genetic and genomic information, sex-determining genes have not been identified in decapod crustaceans; even genes related to sex determination/differentiation have rarely been reported [13]–[15]. Despite the aquacultural and biological importance of *M. nipponense*, previous studies have mostly focused on the isolation of microsatellites [16], [17], the investigation of genetic diversity [18], [19], Sanger-based sequencing of expressed sequence tags (ESTs) [20], the characterization of single functional genes [21]–[23] and sequencing of the mitochondrial genome of *M. nipponense* [1].

**Figure 1:**
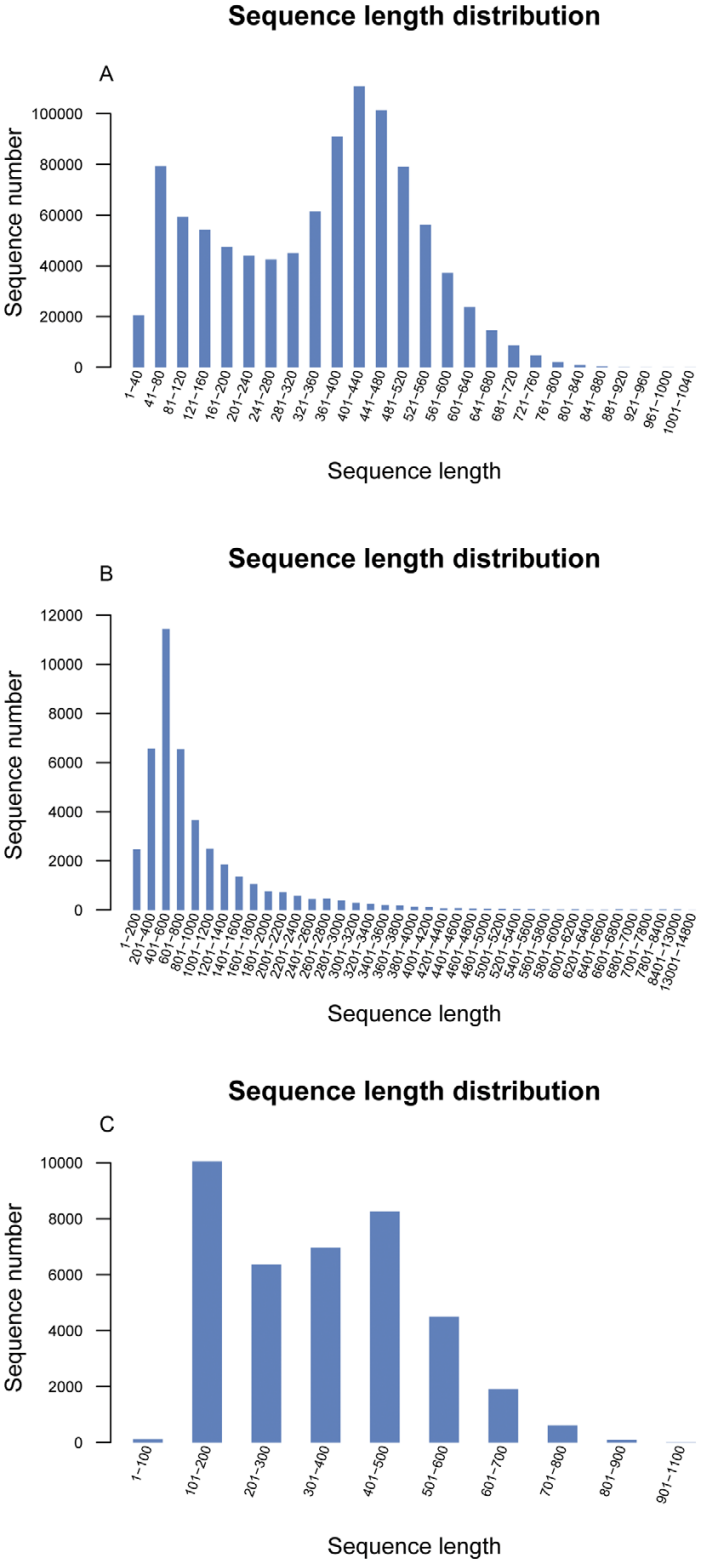
Length distribution of *M. nipponense* transcriptomic ESTs. (**A**) **total transcriptomic reads, (B) contigs, (C) singletons.**

**Table 1:** Summary of Roche 454 GS FLX assembly and analysis of *M. nipponense* transcriptomic sequences.

Dataset name	All
transcriptomic reads	984,204
total number of bases	338,592,850 bp
average read length	344 bp
No. of contigs	42,551
longest contig	14,768 bp
No. of singletons	38,860

It is well known that the generation of large-scale ESTs is a very useful approach to accelerating research in non-model species. ESTs represent a valuable resource for research, as they provide comprehensive information about the transcriptome [24]. ESTs have played significant roles in accelerating gene discovery [25], [26], developing SSRs and SNPs markers [27]–[30], improving genome annotation, facilitating large-scale expression analysis, elucidating phylogenetic relationships, etc. With these useful functions, the number of ESTs in public databases is rapidly increasing. To date, there are more than 72 million ESTs in the NCBI public collection (release 030112; March, 1 2012. http://www.ncbi.nlm.nih.gov/dbEST/dbEST_summary.html). However, less than 8,500 ESTs are available for *M. nipponense*. Transcriptome sequencing enables various functional genomics studies for an organism. Roche 454 Genome Sequencing (GS) FLX technology has generally been used for transcriptome analyses in a large number of plants [24], [27], [31]–[33], animals [29], [30], [34]–[36] and microorganisms [37]. It is particularly useful as a shotgun method for generating EST data, and its costs are relatively low [33], [38]. Moreover, by generating sufficiently long-sequence reads, the 454 GS FLX technology can compensate for the lack of a reference genome during *de novo* sequence assembly [39].

**Figure 2:**
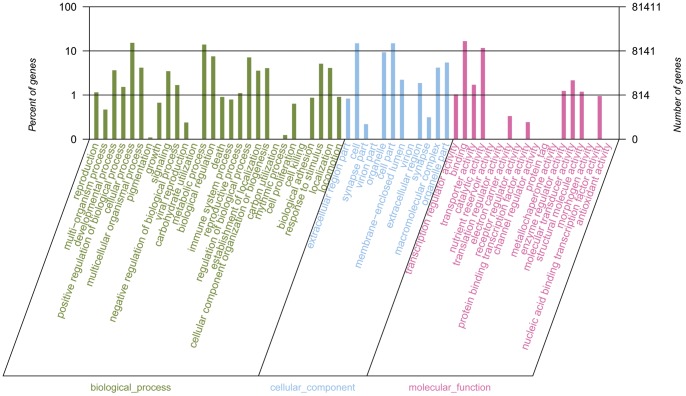
Gene Ontology (GO) terms for the transcriptomic sequences of *M. nipponense*.

**Figure 3:**
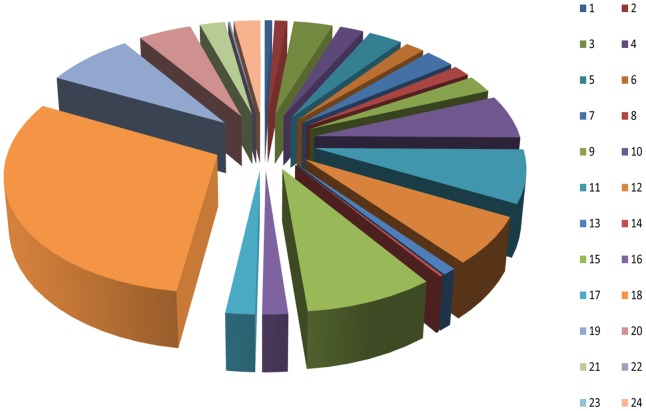
Clusters of orthologous groups (COG) classifications of unique *M. nipponense* sequences. 1: RNA processing and modification; 2: chromatin structure and dynamics; 3: energy production and conversion; 4: cell cycle control, cell division, chromosome partitioning; 5: amino acid transport and metabolism; 6: nucleotide transport and metabolism; 7: carbohydrate transport and metabolism; 8: coenzyme transport and metabolism; 9: lipid transport and metabolism; 10: translation, ribosomal structure and biogenesis; 11: transcription; 12: replication, recombination and repair; 13: cell wall/membrane/envelope biogenesis; 14: cell motility; 15: posttranslational modification, protein turnover, chaperones; 16: inorganic ion transport and metabolism; 17: secondary metabolites biosynthesis, transport and catabolism; 18: general function prediction only; 19: function unknown; 20: signal transduction mechanisms; 21: intracellular trafficking, secretion, and vesicular transport; 22: defense mechanisms; 23: nuclear structure; and 24: cytoskeleton.

In this study, we prepared a cDNA library by pooling total RNA from various organs and tissues, including the eyestalk, gill, heart, ovary, testis, hepatopancreas, muscle, and embryos at the cleavage, gastrula, nauplius and zoea stages. We sequenced ESTs from this library to expand our knowledge of the transcriptome, facilitate future whole-genome sequence assembly in the oriental river prawn, and provide genes for future studies of the molecular mechanisms involved in sex determination/differentiation in *M. nipponense*. This transcriptome dataset provides an invaluable new resource for functional genomics and biological research in *M. nipponense*. The molecular markers identified in this study provide a material basis for future genetic linkage and quantitative trait loci (QTL) analysis and will be essential for accelerating aquaculture breeding programs to increase culture production in the future.

**Table 2:** Selected genes of interest for sex determination/differentiation in the *M. nipponense* transcriptome, including the contigs and singletons.

Candidate genes	Hit(s)	Similarity (%)	E-value	Length (bp)
*Dmrt1*	1	45	3.23E-08	377
*SRY* related	1	33	3.00E-07	807
*FTZ-F1*	4	45	1.84E-08∼4.01E- 08	799∼1330
*SF*	2	60∼88	1.00E-14∼1.77E- 06	318∼404
*P450*	31	56∼90	0∼6.04E-06	157∼1672
*SOX*/HMG-box	9	50∼92	4.01E-99∼4.90E- 09	136∼1521
*FOXL2*	1	50	8.03E-07	619
*ECM*	1	58	0	6049
*FEM1*	8	59∼88	0∼1.12E-08	109∼3831
*STAR*	1	50	5.87E-14	751
*WT1*	1	82	8.80E-134	1164
*GATA*	4	58∼78	5.94E-81∼2.03E- 10	160∼1976
*ZFY1*	4	43∼64	1.00E-16∼1.00E- 11	288∼1127
*WNT*	6	65∼87	4.38E-61∼5.80E- 10	179∼560
*GCL*	2	53∼86	2.16E-08∼6.63E- 06	136∼329
*Argonaute*	6	60∼98	2.65E-156∼3.70E- 13	266∼1968
*Piwi*	2	58∼60	0∼4.18E-133	2625∼3790
*Nanos*	1	74	9.21E-16	391
*Tudor*	11	41∼89	0∼2.70E-07	576∼5692
*Pumilio*	6	52∼83	0∼2.29E-06	127∼6975
*VASA*	1	100	0	2357
*PL10*	1	98	0	5421
testis-specific	11	40∼77	0∼6.48E-07	369∼2524

## Results and Discussion

### Roche 454 GS FLX sequencing and reads assembly

In order to achieve a whole-body *M. nipponense* transcriptome, total RNA was extracted from a variety of adult organs and tissues, including the eyestalk, gill, heart, ovary, testis, hepatopancreas, muscle, and embryos at the cleavage, gastrula, nauplius and zoea stages. Equal quantities of RNA were mixed together to construct a cDNA library. The library was sequenced by a Roche 454 GS FLX.

**Figure 4:**
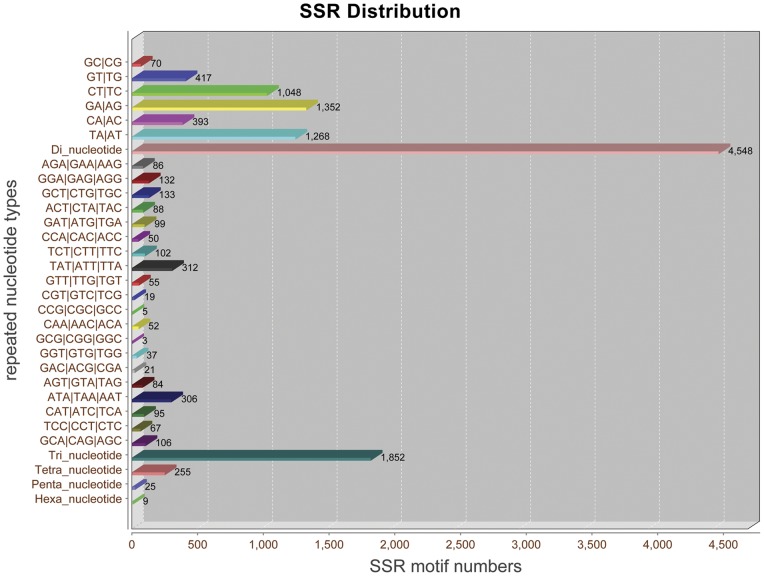
Distribution of simple sequence repeat (SSR) nucleotide classes among different nucleotide types found in the transcriptome of *M. nipponense.* Both contig and singleton sequences are used to predict the SSR loci.

The reads produced by the Roche 454 GS FLX were used for clustering and *de novo* assembly. After eliminating adapter sequences and filtering out the low-quality reads, Roche 454 sequencing yielded a total of 984,204 high-quality transcriptomic reads with a total size of 338,592,850 bp, resulting in an average of 344 bp per read (**Figure 1A**). Clustering and assembly of these reads yielded a non-redundant set of 81,411 ESTs, comprising 42,551 contigs and 38,860 singletons with average lengths of 939 bp and 345 bp, respectively (**Table 1**). Most of these contigs (57.68%) were distributed in the 201∼1000 bp region (**Figure 1B**). And most of these singletons (99.43%) fell between 101 and 800 bp in length (**Figure 1C**). A total of 3,058 contigs were greater than 2.5 kb, with the largest contig at 14,768 bp. The average length of our assembled contigs was longer than that previously reported for *M. rosenbergii* (average of 845 bp) and *Euphausia superba* (average of 492 bp), whose transcriptomes were also obtained with a Roche 454 GS FLX in non-model species [30], [36]. Long sequences of good quality could enable us to obtain more information about genes. Therefore, this transcriptome dataset provides a useful resource for future analyses of genes related to sex determination and sex differentiation. To the best of our knowledge, this is the first comprehensive study of the transcriptome of *M. nipponense*.

**Figure 5:**
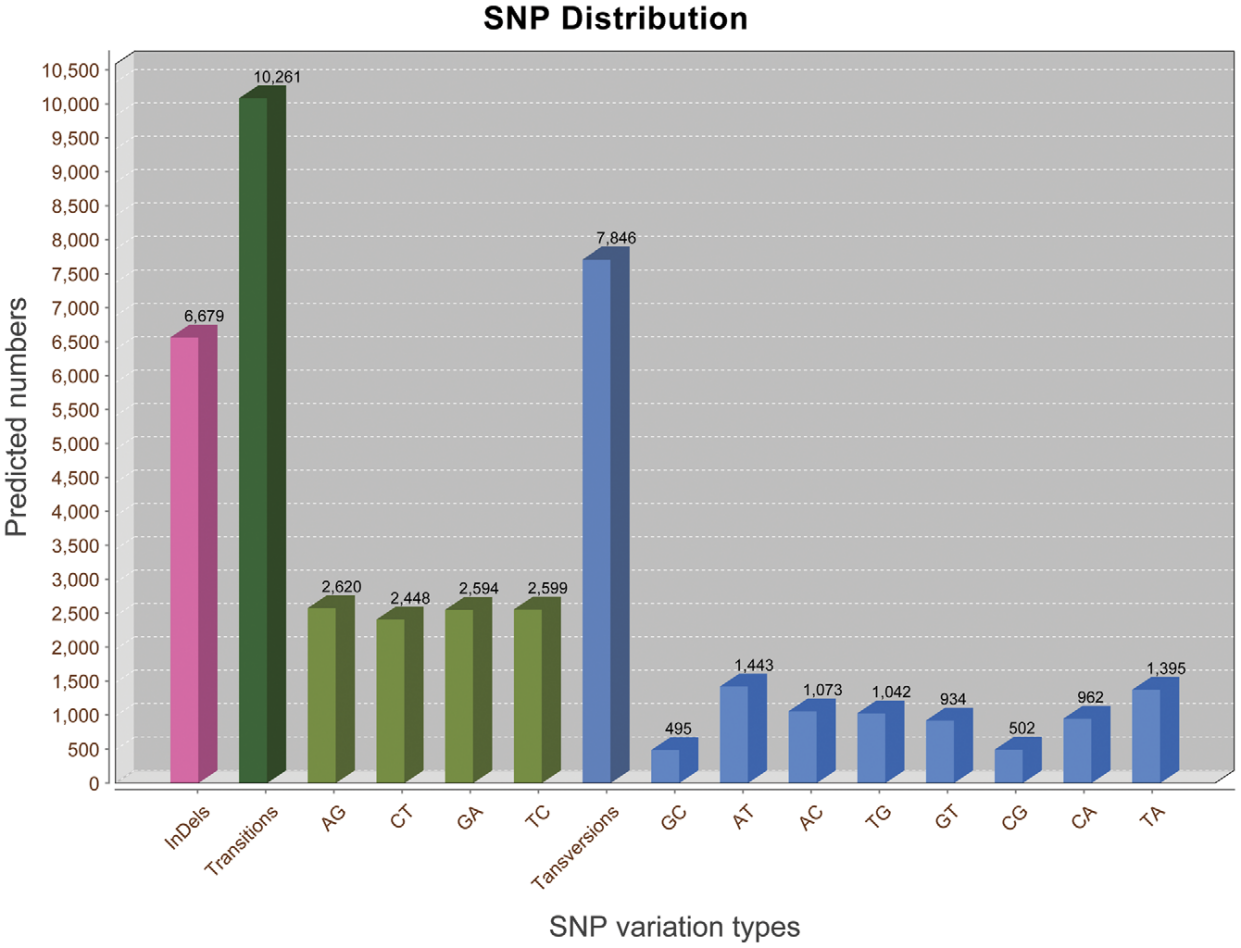
Distribution of putative single nucleotide polymorphisms (SNP) in the transcriptome of *M. nipponense*.

**Table 3:** Primers used and verified SNPs in the ESTs of *M. nipponense*.

Contig ID	Primers sequence (5′–3′)	Products length (bp)	No. of SNPs
06300	AACTTAGGGCTTGCGATTC CTCTTCCGAGTGCGTGAT	262	1/1/0
06559	GTTCTTTGGATGTTAGGTG AAGTTTGTTGATGTGGTTT	221	1/1/0
06567	GACATGACCATGCTCCCA AGGAACTGTCTCGCCAAC	551	0/1/0
07723	ACACTATTTAGGCATTTAT GGAAGTTCAGTAGGAGGT	208	2/2/0
01235	AGCAGACGCAGGTAGAAC AGTGAAGGCTTGAGGAAC	432	1/2/0
01295	GAGCCTCAAATAAGAAAG TATGAAGCCCATCAACCC	431	4/4/0
01191	ACCAGGAATGTTGGAGCA GCCTAGCATACCCGATGA	351	0/5/2
01399	TATCGCATACACTAAGGC ATTTAATGGTCGCTACTC	396	4/5/0
06092	GGAGGAGTGGGTTAGGTC TTACTGTCGCTGTGGTGA	319	2/2/1
08341	ACAGCCATGAAGTCATAAG AGAAGGTCCAGCGATACG	454	3/3/3
08357	GTTATTGCCGTGTTCTTA ATGGTGCTCCCTTATCTT	373	2/3/0
	CAGAAGCACATAGGCACA CAGGTCATCAAGAGGAGG	316	0/2/0
08359	ACTTGCTCCTGATAACCC CCACTTCTTTGACACACC	302	1/1/0
08391	CTCTTCCGCATTTCTACG TAAACCCAGGGATTCAGT	200	1/1/0
08461	TGAATGGGTCTGGTTGGT CATTTGTGGCGGAGGAAG	370	1/1/0
08476	AGTTCACAGCAGTGGGATT TGGTGAGGGAGACGAAGA	200	1/1/0
08491	GGAGAAACGCAAGAACGC TCCTGATGGTGGAGACCC	345	1/1/0
15044	TATTGAAGCTGCACAACC TAGAGCCATAAGCGAGGA	n/a	
27660	TCCGTATCTTTCGAGTTT GTCCCGCATTGTATTTTT	166	1/2/0
08344	GGAAGACAACAACGACAC CCAGCTAACACAAACAAT	496	0/1/0

For each contig, the following are indicated: the number of SNPs verified/the number of SNPs predicted in the fragment/the number of additional SNPs using Sanger technology; n/a indicates that sequencing was not possible because the sequencing signal was interrupted.

### GC content and alternative splicing

The GC content (ratio of guanine and cytosine) of all unique sequences of *M. nipponense* was determined. The concentration of GC was 39.45% and 39.28% in contigs and singletons, respectively, giving rise to an overall GC content of 39.40%, indicating a low GC content in the cDNA of *M. nipponense*.

The *M. nipponense* contigs were further assembled into 38,339 isogroups with an average length of 1,042 bp. More contigs than isogroups were found because some contigs (called isotigs) are attributed to the same isogroups due to alternative splicing. A total of 14.75% of isotigs (6,261 of 42,440) have no less than two methods of alternative splicing. A large number of alternative splicing could improve the utilization rate of the encoding genes. Alternative splicing is an important mechanism for regulating gene expression in eukaryotic cells, and it contributes to protein diversity.

### Comparative analyses of unique sequences

We noted that only 143 (0.18%) unique sequences matched the registered sequences of *M. nipponense* in the GenBank non-redundant database, and 81,298 unique sequences were newly discovered in our present result. This result suggests that we have made a meaningful contribution to the knowledge of *M. nipponense* by characterizing these unique sequences.

Amongst the 81,441 unique sequences, a BLASTx sequencing similarity search of the GenBank nr database matched against 17,342 (40.76%) contigs and 6,882 (17.71%) singletons (with an e-value of 10^−6^) (**Table S1**). Similarly, low matched number of contigs and singletons has been reported in *M. rosenbergii* transcriptomic dataset (46% of contigs and 19% of singletons) using the 454 GS FLX platform [36]. This is due to the lack of genomic information in non-model species. Because *M. nipponense* and *M. rosenbergii* both belong to the genus *Macrobrachium*, they are closely related to one another in taxology. Thus, we found a great number of unique sequences matches (80% sequence similarity) using a BLAST search against the transcriptome of *M. rosenbergii* (data not shown).

A previous report that Roche 454 sequencing of the non-model species, *Mytilus galloprovincialis* produced enough mitochondrial reads to enable alignment of these new data against the relevant mitochondrial sequence [40]. However, this alignment could not be performed with the *M. nipponense* transcriptomic data, as only 10 of the mitochondrial protein-coding genes (PCGs) (cytochrome c oxidase subunit 1, 2 and 3; ATP synthase F0 subunit 6; cytochrome b; and NADH dehydrogenase subunit 1, 2, 3, 4 and 5) were present in our contig dataset. Other PCGs in the *M. nipponense* mitochondrion (ATP synthase F0 subunit 8; and NADH dehydrogenase subunit 4L and 6) were not present in the contig dataset [1]. Singletons potentially contain useful lowly expressed sequences [41]. After searching the singleton dataset, however, we did not find other mitochondrial protein-coding genes. This phenomenon may be because some transcripts with low copies may be lost when constructing non-normalized libraries. The *M. nipponense* transcriptomic library was subjected to many treatments during its construction, and these programmes would contain a substantial proportion of artifacts derived from cDNA synthesis and sequencing to the subsequent reads assembly.

Amongst the unique sequences derived from contigs and singletons, coding sequences with homology to ‘ubiquitin-protein ligase’, ‘ATP synthase’, ‘cell division cycle protein’, ‘cytochrome c oxidase’, ‘cytochrome P450’, ‘zinc finger protein’, ‘NADH dehydrogenase’, ‘pre-mRNA-splicing factor’, ‘serine/arginine-rich splicing factor’ and ‘splicing factor’ were the most abundant. Similarly, most of the transcripts determined in this initial survey were also obtained in the *M. rosenbergii* transcriptomic dataset [42]. Although our research mainly focused on finding putative genes related to sex determination and sex differentiation, other putative functional transcripts identified here could provide a foundation for future investigations of the roles of stress response, reproduction and defense reaction. The transcriptomic findings could also be the best source for deciphering the putative functions of novel genes, but further studies would need to be conducted to understand their molecular functions.

### Gene Ontology assignments

Gene Ontology (GO) could provide a structured and controlled vocabulary for describing gene products in three categories: molecular function, cellular component and biological process [43]. We added GO terms using Blast2GO [44], which is based on the automated annotation of all unique sequences in the GenBank nr protein database from NCBI. According to the database, a total of 19,092 unique *M. nipponense* sequences were assigned to one or more ontologies based on their similarity to sequences with previously known functions, including 30,425 sequences assigned to the molecular function category, 44,112 to the cellular component category and 67,679 to the biological process category. The assigned sequences were divided into 58 functional terms (**Table S2**). Because several of the sequences were assigned to more than one GO term, the total number of GO terms obtained in our dataset was much bigger than the total number of the unique sequences.

In the molecular function category, ‘binding’, ‘catalytic activity’, ‘molecular transducer activity’, ‘transporter activity’ and ‘enzyme regulator activity’ comprised the largest proportion, accounting for 89.47% of the total (**Figure 2**). Whilst the cellular component category showed that many unique sequences were to likely possess ‘cell’ (27.46%), ‘cell part’ (27.46%) and ‘organelle’ (17.19%) functions. Moreover, the top five biological functions assigned were involved in ‘cellular process’ (18.33%), ‘metabolic processes’ (16.74%), ‘biological regulation’ (9.07%), ‘regulation of biological process’ (8.57%) and ‘response to stimulus’ (6.17%) (**Table S2**). In summary, these terms account for a large fraction of the overall assignments in *M. nipponense* transcriptomic dataset. Understandably, genes encoding these functions may be more conserved across different species and are thus easier to annotate in the database.

### KEGG analysis and COG analysis

The unique sequences were mapped to the KEGG database to define metabolic pathways. According to the KEGG results, 42,126 unique sequences, comprising 39,302 contigs and 2,824 singletons, were mapped onto 123 and 90 predicted KEGG metabolic pathways, respectively (**Table S3**). In order to perform a comprehensive KEGG study, we added the unique sequences derived from singletons to our analysis. Ultimately, we found one type of metabolic pathway, ‘bisphenol degradation’, which did not include in the contigs.

The numbers of unique sequences mapped to various pathways ranged from 1 to 11,667. The main metabolic pathways of unique sequences in the *M. nipponense* transcriptome are ‘purine and pyrimidine metabolism’, ‘amino acid metabolism’, ‘fatty acid metabolism’ and ‘carbohydrate metabolism’. As might be expected, some putative enzymes belonging to arthropod were found in the KEGG analysis of amino sugar and nucleotide sugar metabolism. The enzymes ‘chitin synthase’ and ‘chitinase’ play an important role in carapace formation and exuviation during the molting cycle [45], [46]. Considering the growth process habit of *M. nipponense*, finding these pathways in the dataset was not surprising.

Assignments of COG were used to predict and classify possible functions of the unique sequences. Based on sequence homology, 3,114 unique sequences had a COG functional classification. These sequences were classified into 24 COG categories (**Figure 3**). The most common category was ‘general function prediction only’ with 938 unique sequences. This category was followed by ‘posttranslational modification, protein turnover, chaperones’ (264) and ‘function unknown’ (248). ‘Nuclear structure’ (2), ‘defense mechanisms’ (3) and ‘cell motility’ (7) were the smallest COG categories.

KEGG pathway analysis and COG analysis are helpful for predicting potential genes and their functions at a whole-transcriptome level. The predicted metabolic pathways, together with the COG analysis, are useful for further investigations of gene function in future studies.

### Genes of interest related to sex determination

The transcriptome of *M. nipponense* was primarily examined to identify a wide range of candidate genes that might be functionally associated with sex determination and sex differentiation. Traditionally, such gene discovery in non-model organisms has required degenerate PCR, which is labor-intensive and prone to failure [47]. The annotated transcriptome reported here allows researchers to identify genes of interest more easily than that of using degenerate PCR. According to our sequence analysis and published literature, many genes involved in sex determination and sex differentiation, including sex-determining region Y-chromosome (*SRY*), doublesex- and mab-3-Related transcription factor 1 (*DMRT1*), fushi tarazu factor 1 (*FTZ-F1*), forkhead box L2 (*FOXL2*), feminization 1 (*FEM1*), *VASA* and other potential candidates, were identified (**Table 2**).

In mammals, the male sex determination switch is controlled by *SRY* [48]. One unique sequence similar to *SRY* was identified in the *M. nipponense* transcriptome, displaying 33% similarity with homologues in mouse. *SRY* has DNA-binding properties that are mediated by a high mobility group (HMG-box) motif [49], named *SOX* [50]. After searching the transcriptomic dataset, three unique sequences were annotated as *SOX2*, *SOX14* and *SOX19*. The sequences exhibited significant BLAST e-values (8.92E-67, 6.74E-61 and 3.61E-64) with those of *Pediculus humanus corporis*, *Scylla paramamosain* and *Tribolium castaneum*, respectively. Whilst two unique sequences were identified as HMG-box a and HMG-box b and displayed significant similarities (83% and 92%) with the corresponding sequences in *Litopenaeus vannamei*.

*DMRT1*, a gene related to worm and fly sexual regulators, is required for mammalian testis differentiation [51]. In all birds, *DMRT1* is located on the Z chromosome but is absent from the W. In chicken embryos, it is expressed in the early bipotential gonad and shows higher expression in males than in females [52]. Knockdown of *DMRT1* in early chicken embryos can result in the feminization of male gonads [53]. One unique sequence was aligned as *DMRT1*, displaying a BLAST e-value of 3.23E-08 and 45% similarity to the encoded amino acid sequence of homologues in *Pan troglodytes*. *FOXL2* is a member of the forkhead/HNF-3-related family of transcription factors involved in ovarian differentiation [54]. Recent research has shown that *FOXL2* suppresses testicular differentiation mainly through repression of the *SOX9* regulatory element, which promotes testis-specific expression of *SOX9* and leads to gonadal sex reversal in mice [55]. Likewise, one unique sequence, which was identified as *FOXL2*, exhibited 50% similarity in its encoded amino acid sequence with homologues in *Homo sapiens* as well as that of *DMRT1*. Lower sequence similarity and less quantity of the transcript were interpreted as a consequence of earlier genetic differentiation between phyla. Meanwhile, *DMRT1* and *FOXL2* might not be the dominant sex determination/differentiation genes in *M. nipponense*.

*FTZ-F1* is a member of the nuclear receptor superfamily and was originally found as a regulator of the *Drosophila* homeobox segmentation gene *FTZ* [56]. In mammals, *FTZ-F1* is an essential factor in sex differentiation. To date, its homologues have been identified in human, mouse and a number of teleost species [57]–[60]. Four unique sequences were annotated as *FTZ-F1* with *Apis mellifera FTZ-F1*. However, no studies about *FTZ-F1* have been published for *Macrobrachium*.

*FEM1*, which is considered as a signal-transducing regulator, was first reported in the *Caenorhabditis elegans* sex determination pathway. *FEM1* of *C. elegans* plays a global role in sex determination. Nevertheless, a single *FEM1* transcript and protein are expressed at equivalent levels in both sexes, suggesting primarily posttranscriptional and posttranslational regulation of its activity [61]. Its homologs, including *FEM1A*, *FEM1B* and *FEM1C* of sex determination for candidate genes have been found in human and house mouse (*Mus musculus*). In *M. nipponense*, 11 unique sequences were identified as *FEM1A*, *FEM1B* and *FEM1C*, with similarities ranging from 49%∼93%, respectively. To the best of our knowledge, these putative *FEM1A*, *FEM1B* and *FEM1C* genes are first reported in crustacean.

*VASA*, belonging to the DEAD-box family of ATP-dependent RNA helicase, plays a crucial role in germ cells development [62]. Its product is required maternally for germ plasm assembly and completion of oogenesis. Since *VASA* is expressed specifically in germ cells of most animals, it was used as a reliable molecular marker to trace the origin, migration, and differentiation of primordial germ cells [63], [64]. In the *M. nipponense* transcriptome, one unique sequence was aligned with *VASA*, displaying 100% similarity in encoded amino acid sequence with homologues in *M. nipponense* which amino acid sequence was previously submitted to NCBI database by our laboratory. This similarity suggests that the *M. nipponense* transcriptome presented here is accurate and can be trusted.

In this study, we also identified other putative genes related to sex determination/differentiation in *M. nipponense* for the first time, such as cytochrome P450 aromatase *(P450*) [65], steroidogenic acute regulatory protein (*STAR*) [66], Zinc finger Y-chromosomal protein 1 (*ZFY1*) [67], Wilms' tumor protein 1 (*WT1*) [68] and *Tudor* [69]. The research described herein provides no evidence for a master gene whose presence or absence determines *M. nipponense* sex or sex differentiation. Some studies showed that in *M. rosenbergii*, as in other macruran species, the female prawn is the heterogametic sex, bearing WZ sex chromosomes, whereas the male prawn is the homogametic sex, bearing ZZ sex chromosomes [8], [12], [70]. While *M. nipponense* belongs to the same genus as *M. rosenbergii*, it is uncertain whether *M. nipponense* possesses the same sex determination mechanism. Further studies are needed to understand the molecular functions of these putative genes. Sex determination and sex differentiation are very complicated processes. Understanding gene regulatory mechanisms has not only theoretical value, but also practical value and meaning for aquaculture.

### Putative molecular markers

SSRs, or microsatellites, are polymorphic loci present in genomic DNA. They consist of repeated core sequences of 2∼6 base pairs in length. Among the various molecular markers, SSRs have been proven to be an efficient tool for constructing genetic linkage, performing QTL analysis and evaluating the level of genetic variation in a species because of the high variability, abundance, neutrality and co-dominance of microsatellite DNA [71].

We obtained a total 6,689 SSRs in the transcriptomic dataset. Of these, 67.99% were di-nucleotide repeats, followed by 27.69% tri-nucleotide repeats and 4.32% tetra/penta/hexa-nucleotide repeats (**Figure 4**). Among the di-nucleotide repeats motifs, (GA/AG)_n_, (TA/AT)_n_ and (CT/TC)_n_ were the three predominant types with frequencies of 29.73%, 27.88% and 23.04%, respectively. There was a bias towards tri-nucleotide repeats motifs composed of A and T. In the 20 types of tri-nucleotide repeats, (TAT/ATT/TTA)_n_ and (ATA/TAA/AAT)_n_ were the most common types with a combined frequency of 33.37%. To date, only a few microsatellites have been available for *M. nipponense* from NCBI. Thus, the development of SSRs for this species is highly desirable.

SNPs were identified from alignments of multiple sequences used for contig assembly. By excluding those that had mutation frequency of bases lower than 1%, we obtained a total of 24,786 SNPs, of which 6,679 were putative indels (In), 10,261 were putative transitions (Ts) and 7,846 were putative transversions (Tv), giving a mean In: Ts: Tv ratio of 1∶1.54∶1.17 across the transcriptome of *M. nipponense* (**Figure 5**). The AG/GA, CT/TC and AT/TA SNP types were the most common. In contrast, GC/CG types were the smallest SNP types because of the differences in the base structure and the number of hydrogen bonds between different bases. Multiple sequence alignment also identified a total of 6,679 indels across the transcriptome. It should be treated with caution because of technical problems associated with Roche 454 GS FLX pyrosequencing [32]. To verify the potential SNPs, a subset of 20 ESTs containing 39 SNP loci was selected randomly. A pooled cDNA sample of eight wild *M. nipponense* was amplified by PCR. Subsequently, PCR products were sequenced bidirectionally with forward or reverse primers (**Table 3**). Of the 39 SNP loci predicted to reside in the amplified sequences, 26 (66.67%) showed polymorphisms in the sample and were validated (**Table 3**). The rate of polymorphic SNPs was probably an underestimate because only eight individuals were used. Consequently, using more *M. nipponense* samples, the polymorphic rate of potential EST-SNPs should be higher than that found in our validation.

Six additional SNPs, which were not detected in the transcriptomic dataset, were identified by genotyping 20 ESTs (**Table 3**). This phenomenon of missing SNPs may be due to the experimental methodology. Because 454 pyrosequencing was used to randomly sequence cDNAs, the detection of polymorphisms was likely hindered by the requirement of at least two reads with the variant alleles. Moreover, these reads had to contain at least 20 nucleotides of conserved sequence upstream and downstream of the locus.

SSRs and SNPs detected in this study (**Table S4**) are likely to be highly transferable to other closely related species, as has been the case in other crustacean species [72], [73]. We also envision that these potential markers identified within the ESTs will be valuable for studying the evolution and molecular ecology of *Macrobrachium* species, genome mapping, and QTL analysis.

### Conclusions

In this study, *de novo* transcriptome sequencing for *M. nipponense* using the 454 GS FLX was performed for the first time. A total of 984,204 high-quality transcriptomic reads were obtained, giving rise to an average of 344 bp per read. A significant number of putative metabolic pathways and functions associated with the unique sequences were identified. Moreover, a large number of SNPs and SSRs were predicted and can be used for subsequent marker development, genetic linkage and QTL analysis. Many candidate genes that are potentially involved in sex determination and sex differentiation were identified for the first time and are worthy of further investigation. Our study provides the largest number of ESTs to date and lays the initial groundwork for in-depth, functional transcriptomic profiling of *M. nipponense*.

## Materials and Methods

### Ethics statement

Collecting the wild oriental prawns from Chongming Island (Shanghai, China) was permitted by the Department of Fishery of Chongming County (permit number 2011HY000108). All handling of prawns were conducted in accordance with guidelines on the care and use of animals for scientific purposes set up by the Institutional Animal Care and Use Committee (IACUC) of Shanghai Ocean University, Shanghai, China.

### Tissue material, RNA extraction and quality controls

Adult *M. nipponense* prawns (nine males and six females) used in this study were collected from Chongming Island (Shanghai, China) in 2011. The prawns were all anaesthetized on ice and dissected to collect samples, including the eyestalk, gill, heart, ovary, testis, hepatopancreas, muscle, and embryos at the cleavage, gastrula, nauplius and zoea stages. All of the samples were immediately frozen in liquid nitrogen and stored at −80°C until use. Total RNA was extracted from these materials using TRIzol Reagent (Invitrogen, USA) according to the manufacturer's instructions. The quality of total RNA was determined using a Nanodrop spectrophotometer (Thermo, USA). Only RNA samples with a 260 of 280 ratio from 1.9 to 2.1 and a 260 of 230 ratio from 2.0 to 2.5 were used for the next analysis.

### Library construction and 454 pyrosequencing

We combined equivalent amounts of total RNA from each sample (∼0.20 mg total RNA) and delivered it to Shanghai Majorbio Bio-pharm Biotechnology Co., Ltd. (Shanghai, China) for the construction of the cDNA library. The cDNA library was constructed by the SMART cDNA library construction kit (Clontech, USA) following the manufacturer's protocol step-by-step. cDNA was sheared by nebulization and DNA bands (500∼800 bp) were extracted after agarose gel electrophoresis. Then, the obtained cDNA was purified, blunt ended, ligated to adapters and finally small fragments were removed. The quality control of a double DNA library was performed using High Sensitivity Chip (Agilent Technologies). The concentration was examined by TBS 380 Fluorometer. After the examination, one-plate, whole-run sequencing was performed on Roche 454 GS FLX Titanium chemistry (Roche Diagnostics, Indianapolis, IN, USA) by Shanghai Majorbio Bio-pharm Biotechnology Co., Ltd. following the manufacturer's protocol.

### Sequence cleaning and assembly

For each sequence, low-quality bases and the sequencing adapter were trimmed using LUCY (http://lucy.sourceforge.net/) and SeqClean (http://compbio.dfci.harvard.edu). The remained sequencing reads were first assembled using the Newbler software with the “extend low depth overlaps” parameter. In order to improve the assembler quality, we collected all available ESTs of *Macrobrachium* from NCBI-EST database (http://www.ncbi.nlm.nih.gov/nucest?term=macrobrachium). All of the ESTs from the Roche 454 and NCBI-EST databases were used to run the final assembly of *M. nipponense*.

### Annotation of mRNAs

BLASTx searches [74] of the GenBank nr database hosted by NCBI (http://www.ncbi.nlm.nih.gov/) were performed on all unique sequences to identify the putative mRNA functions (e-value threshold ≤1e^−6^, e values less than 1.0×10^−6^ were considered significant). Additionally, GO terms (http://www.geneontology.org) were extracted from the best hits obtained from the BLASTx against the nr database using Blast2GO. These results were then sorted by GO categories using in-house Perl scripts. BLASTx was also used to align unique sequences to the Swiss-Prot database (http://web.expasy.org/docs/swiss-prot_guideline.html), Kyoto Encyclopedia of Genes and Genomes (KEGG) and Clusters of Orthologous Groups (COG) (http://www.ncbi.nlm.nih.gov/COG/) (with the e-value of 10^−6^) to predict possible functional classifications and molecular pathways [75], [76].

### Identification of EST-SSR motifs and EST-SNPs

The unique sequences were screened for microsatellites using the Mreps software (http://bioinfo.lifl.fr/mreps/) with default parameters. Perfect di-, tri-, tetra-, penta-, and hexa-nucleotide motifs were detected, and all SSR types required a minimum of 6 repeats.

The SNPs were extracted using VarScan (http://varscan.sourceforge.net) with the default parameter only when both alleles were detected from the contigs. Since no reference sequences were available, SNPs were identified as superimposed nucleotide peaks where two or more reads contained polymorphisms at the variant allele. To validate the putative SNPs identified in ESTs, a cDNA pool of eight wild *M. nipponense* were used. Twenty ESTs containing 39 potential SNP loci and sufficient flanking regions were randomly selected for primer design to amplify 150∼600 bp of fragments. PCR products were sequenced directly in both directions with forward and reverse primers using Sanger technology on the ABI3730 platform (Applied Biosystems). Sequencing chromatograms were visually analyzed with Chromas 2.32 (Technelysium Pty. Ltd.), and SNPs were identified as overlapping nucleotide peaks.

### Data Deposition

The Roche 454 reads of *M. nipponense* were submitted to NCBI Sequence Read Archive under the accession number of SRA051767.2.

## Supporting Information

Table S1 **Summary of BLASTx results for contigs and singletons of** ***M. nipponense*** **.**
(XLS)

Table S2 **Categories of Gene Ontology of** ***M. nipponense*** **unique sequences.**
(XLS)

Table S3 **KEGG summary of** ***M. nipponense*** **unique sequences.**
(XLS)

Table S4 **Summary of putative SSRs and SNPs from** ***M. nipponense*** **transcriptome.**
(XLS)
